# Mediating factors associated with alcohol intake and periodontal condition

**DOI:** 10.3389/froh.2025.1524772

**Published:** 2025-04-24

**Authors:** Yuto Kusu, Michiko Furuta, Shinya Kageyama, Yoshihisa Yamashita, Toru Takeshita

**Affiliations:** ^1^ Section of Preventive and Public Health Dentistry, Division of Oral Health, Growth and Development, Faculty of Dental Science, Kyushu University, Fukuoka, Japan; ^2^ Kyushu Dental University, Kitakyushu, Japan

**Keywords:** alcohol consumption, periodontal disease, obesity, hyperglycemia, liver abnormality

## Abstract

**Background:**

Alcohol consumption has been reported to increase the risk of periodontal disease and various health abnormalities such as obesity, hyperglycemia, and liver abnormalities. While the link between these health abnormalities and periodontal disease has been established, their potential mediating role in the association between alcohol consumption and periodontal disease remains unclear. Therefore, this study aims to investigate the multiple mediating roles of obesity, hyperglycemia, and liver abnormalities in this association.

**Methods:**

A cross-sectional study was conducted on 6,529 individuals aged 35–64 years who underwent workplace health check-ups in 2003 (mean age: 45.7 ± 8.7 years). The periodontal condition was evaluated using the mean pocket depth (PD), and participants were classified into no, light/moderate (alcohol consumption 0.1–29.9 g/day), and heavy (≥30 g/day) drinking groups. Causal mediation analysis was performed.

**Results:**

Heavy drinking had a direct effect on the mean PD. Light/moderate drinking had a indirect effect on the mean PD through the body mass index (BMI), glucose level, alanine aminotransferase level (ALT), with proportion mediated of 25.1%, 8.9%, and 18.9%, respectively. The mediating role of glucose level was found in the association between heavy drinking and the mean PD with proportion mediated of 32.7%.

**Conclusion:**

This study confirmed that alcohol consumption was associated with worse periodontal condition among Japanese adults who received workplace health check-ups. This association was partially contributed by several factors such as BMI, glucose level, and ALT.

## 1 Introduction

High alcohol consumption has been demonstrated to increase the risk of several chronic disorders such as obesity, diabetes, hypertension, and liver disease (1). From 1990 to 2017, the global alcohol consumption per capita increased from 5.9 L to 6.5 L, and it is estimated to further increase by 17%, reaching 7.6 L in 2030 (2). Along with increased alcohol consumption at the global level, a considerable increase in the alcohol-associated health risks of adults is expected (1).

One such health risk is periodontal disease, which has been shown to have an association with high alcohol consumption (3, 4). This association may be caused by the direct effect of alcohol on alveolar bone loss, with inflammatory infiltrate, osteoclastogenesis, tumor necrosis factor-α (TNF-α) production, and increased gingival oxidative stress observed in periodontal tissue (5–7). Additionally, alcohol may indirectly impact periodontal disease through systemic health deterioration such as obesity, diabetes, and liver abnormalities (8).

Alcohol adds energy to a meal and stimulates food intake, which leads to obesity (9). In turn, the chronic and systemic low-grade state of inflammation observed in obese individuals may induce periodontal disease through the secretion of inflammatory factors from adipose tissue, increasing periodontal inflammation and promoting bacterial proliferation (10). Moreover, heavy drinking has a direct toxic effect on the pancreatic islet cells and can subsequently cause diabetes (11). Diabetes can also lead to periodontal disease through increased inflammation in periodontal tissues due to exacerbated and dysregulated inflammatory responses (12–14). Similarly, alcohol can cause liver injury through direct toxic effects (15), consequently affecting periodontal disease by increasing the levels of pro-inflammatory cytokines and reducing the levels of anti-inflammatory cytokines (16).

These previous studies suggest the possibility that alcohol consumption may be associated with periodontal disease through obesity, diabetes, and liver abnormalities. However, to our knowledge, the mediation effect of these health abnormalities on the association between alcohol consumption and periodontal disease has not previously been studied. Therefore, this study aimed to investigate the direct association between alcohol consumption and periodontal disease and the mediating role of obesity, diabetes, and liver abnormalities.

## 2 Materials and methods

### 2.1 Study population

**Figure 1:**
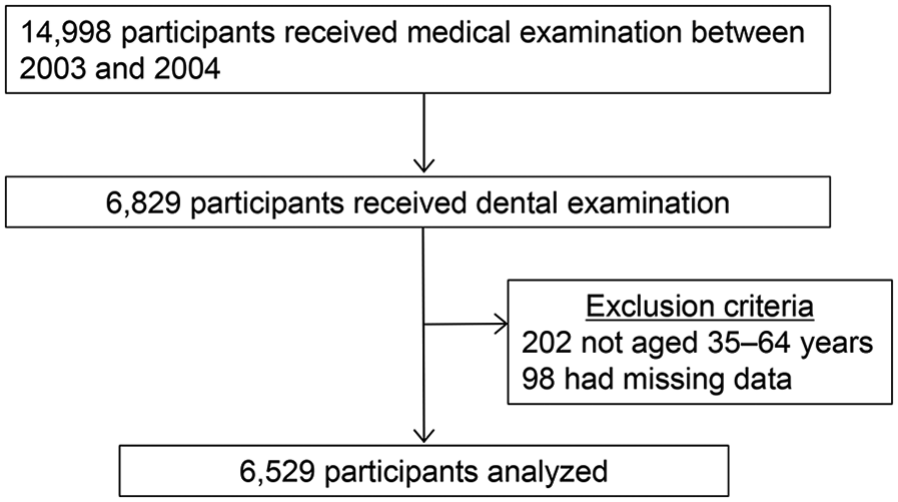
Flow diagram of the study participants.

This cross-sectional study was conducted at the health care center of a manufacturing company in Yokohama, Japan, where regular health examinations, including dental check-ups, were conducted to assess the health statuses of employees and their families. In total, 14,998 employees underwent medical examination between 2003 and 2004; of these, 6,829 individuals opted in for and received dental examination (17). After excluding patients not aged 35–64 years (*n* = 202) and those with missing values (*n* = 98), a total of 6,529 participants were selected for inclusion in the analyses (Figure 1).

This study was approved by the Institutional Review Board of the Faculty of Dental Science, Kyushu University, Fukuoka, Japan (approval no. 19B-5). Informed consent was obtained from all participants. This study follows the STROBE guidelines for observational studies.

### 2.2 Oral examination

Oral health status was assessed by the number of present teeth and the periodontal condition. These oral examinations were performed by 10 trained and calibrated dentists, using a procedure as described elsewhere (18). One experienced dentist served as the gold-standard examiner (YS). The intra-class correlation coefficient was used to measure inter-examiner agreement with the gold standard. This was 0.839 and 0.840 for pocket depth (PD) and clinical attachment level (CAL).

Periodontal examination was performed according to the Third National Health and Nutrition Examination Survey III (NHANES III) method at two sites (buccal proximal and central) on all teeth except the wisdom teeth. Periodontal status was evaluated by PD and CAL, with the mean PD and CAL calculated as the sum of the maximum PD per tooth divided by the number of teeth present for each individual. We classified periodontal disease according to the 1999 case definition, with moderate periodontal disease characterized as the presence of two or more interproximal sites with a CAL ≥4 mm, not on the same tooth, or two or more interproximal sites with a PD ≥5 mm, not on the same tooth (19). Furthermore, severe periodontal disease was characterized as the presence of two or more interproximal sites with a CAL ≥6 mm, not on the same tooth, or one or more interproximal sites with a PD ≥5 mm, not on the same tooth (19).

### 2.3 Medical examination

Anthropometric measurements of height and weight were taken and body mass index (BMI) was calculated. Obesity was defined as BMI ≥25.0 kg/m^2^, which was the optimal cut-off for obesity in Asian individuals (20). Additionally, plasma concentrations of fasting glucose, alanine aminotransferase (ALT), and aspartate aminotransferase (AST) were determined from blood samples, while ultrasonography was performed by gastroenterological specialists to check for fatty liver according to standardized criteria (hepatorenal echo contrast, liver brightness, deep attenuation, and vascular blurring) (21). Elevated fasting glucose level was defined as ≥100 mg/dl (22). Abnormal ALT level was determined by ≥30 IU/L in males and ≥19 IU/L in females, and abnormal AST level was defined as ≥35 IU/L (23).

### 2.4 Questionnaire

Alcohol consumption, smoking, regular dental visits, and toothbrushing frequency were investigated using a questionnaire. This questionnaire assessed alcohol consumption using questions regarding the type, amount, and frequency of alcohol consumed. The daily alcohol intake was calculated by multiplying the number of drinking days per week by the ethanol amount and dividing the sum by seven. This daily alcohol intake was divided into the following three groups: no (0 g/day), light/moderate (0.1–29.9 g/day), and heavy (≥30 g/day) drinking groups (24, 25). Furthermore, smoking was categorized as current or not current, the regularity of dental visits was categorized as regular or not regular, and the toothbrushing frequency was categorized as “twice or less a day” or “three or more times a day”.

### 2.5 Statistical analysis

Descriptive statistics were employed to assess the periodontal condition, alcohol consumption, medical condition, and health behaviors, with continuous values presented as mean ± standard deviation and categorical values expressed as numbers and percentages. Fasting glucose, ALT, and AST were natural log-transformed to normalize their skewed distribution. Univariate analysis was performed to identify the factors related to the periodontal condition using linear regression analysis.

The directed acyclic graph (DAG) was conducted to identify the minimum set of covariates in the multivariable models using the CAUSALGRAPH procedure in SAS version 9.4 (SAS Institute, Cary, NC). The DAG representing alcohol consumption as the exposure variables, BMI, fasting glucose, ALT, and fatty liver as mediators, and periodontal condition as outcome was illustrated in Supplementary Figure S1. Each mediator was separately included in the DAG model. The covariates were age, sex, smoking, regular dental visits, toothbrushing frequency, and the number of present teeth. The multivariable model included the variables identified by the DAG.

**Figure 2:**
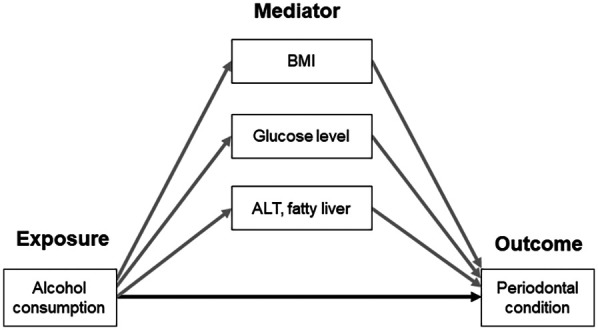
Conceptual model of the association between alcohol consumption and periodontal condition, mediated by BMI, glucose level, ALT, and fatty liver. Gray- and black-colored arrows indicate the indirect and direct association, respectively. ALT, alanine aminotransferase; BMI, body mass index.

Moreover, the causal mediation analysis based on the potential outcomes framework and the more general counterfactual framework (26) was performed to investigate the mediating effects of BMI, fasting glucose, ALT, and fatty liver on the association between alcohol consumption and the periodontal condition, represented by the mean PD and CAL or severe periodontal disease (Figure 2). The causal mediation analysis can decompose the total effect into direct and indirect effects (natural direct effect, NDE and natural indirect effect, NIE, respectively) even in the presence of exposure-mediator interactions, which is a limitation of traditional mediation methods (26). NDE represents the change in outcome that would be observed if the exposure were changed while keeping the mediators unchanged. NIE represents the change in the outcome that occurs due to variations in the mediator when the exposure influences the mediator, while holding the exposure itself constant (26). The proportion mediated which was interpreted as the percentage of the main association that can be explained by the mediator. The proportion mediated was calculated by dividing the indirect effect by the total effect when the indirect effects were significant. The mediators included in this study were BMI, fasting glucose, ALT, and fatty liver, and each mediator was separately included in the causal mediation model. The confidence interval for the proportion mediated was not estimated because it is highly variable, and NIE is recommended to assess the significance of mediation effect (27). The causal mediation models were also tested when the mediators were categorical (i.e., obesity, elevated fasting glucose, abnormal ALT and AST levels). All mediation models included age, sex, smoking, regular dental visits, toothbrushing frequency, and the number of present teeth as covariates.

All statistical analyses except DAG were performed using Stata SE version 18.0 (Stata Corp LP, College Station, TX, USA), with the causal mediation analysis implemented using the *mediate* package. All tests were two-tailed, with statistical significance set at *p* < 0.05.

## 3 Result

### 3.1 Baseline characteristics

The characteristics of participants are shown in Table 1. Notably, the mean PD value was 2.25 ± 0.50 mm, and the proportions of participants in the no-drinking, light/moderate-drinking, and heavy-drinking groups were 20.6%, 63.3%, and 16.1%, respectively.

**Table 1:** Characteristics of the study population.

Variables	Study population
	(*n* = 6,537)
Mean PD, mm	2.25 ± 0.50
Mean CAL, mm	2.54 ± 0.74
Periodontal disease	
No/mild	4,040 (61.8%)
Moderate	2,183 (33.4%)
Severe	314 (4.8%)
Alcohol consumption	
No	1,344 (20.6%)
Light/moderate	4,135 (63.3%)
Heavy	1,050 (16.1%)
BMI, kg/m^2^	23.0 ± 3.2
Obesity (BMI ≥25.0)	1,525 (23.3%)
Fasting glucose, mg/dl*	97.5 ± 16.3
Elevated fasting glucose (≥100)*	1,931 (29.5%)
ALT, IU/L	20.2 ± 9.3
Elevated ALT (males ≥30, females ≥19)	1,607 (24.6%)
AST, IU/L	23.6 ± 17.7
Elevated AST (≥35)	326 (5.0%)
Fatty liver	1,513 (23.1%)
Age	45.7 ± 8.7
Gender	
Males	5,030 (76.9%)
Female	1,507 (23.1%)
Number of present teeth	27.8 ± 2.6
Toothbrushing (daily frequency)	
≥3 times	1,227 (18.8%)
≤2 times	5,310 (81.2%)
Regularity of dental check-ups	
Irregular	5,817 (89.0%)
Regular	720 (11.0%)
Current smoking	
No	4,825 (73.8%)
Yes	1,712 (26.2%)

* Missing value (*n* = 8).

Continuous variable expressed as mean ± SD; categorical variables, as *n* (%).

PD, pocket depth; CAL, clinical; attachment level; BMI, body mass index; ALT, alanine aminotransferase; AST, aspartate aminotransferase.

### 3.2 Univariate analysis

Table 2 presents the results of univariate analysis of the association between risk factors and mean PD. Notably, alcohol consumption, BMI, fasting glucose, ALT, and fatty liver were positively associated with the mean PD.

**Table 2:** The univariate analysis of factors associated with mean PD.

Variables	β	SE	*p* value
Alcohol consumption (ref. no)			
Light/moderate	0.047	0.016	0.003
Heavy	0.150	0.021	<0.001
BMI	0.021	0.002	<0.001
Fasting glucose^†^	0.444	0.046	<0.001
ALT^†^	0.072	0.011	<0.001
AST^†^	0.100	0.019	<0.001
Fatty liver (ref. no)	0.070	0.015	<0.001
Age	0.009	0.001	<0.001
Female (ref. male)	−0.141	0.015	<0.001
Number of present teeth	−0.034	0.002	<0.001
Toothbrushing frequency of ≤2 times a day (ref. ≥3 times)	0.109	0.016	<0.001
Irregular dental visit (ref. regular)	0.061	0.020	0.002
Current smoking (ref. no)	0.137	0.014	<0.001

^†^ Log-transformed values were used.

The crude model of linear regression analysis included the mean PD as the outcome and each associated factor as the independent variable.

PD, pocket depth; β, β-coefficient; SE, standard error; BMI, body mass index; ALT, alanine aminotransferase; AST, aspartate aminotransferase.

### 3.3 Mediation analysis

Table 3 shows the results of causal mediation analysis, which decomposed the total effect of alcohol consumption on mean PD into direct and indirect effects through three potential mediators such as BMI, fasting glucose, and ALT. Heavy drinking directly increased mean PD in Models 1 and 3. In Model 1, which includes BMI, NDE was β = 0.061, standard error [SE] = 0.024, and *p* = 0.009. In Model 3, which includes ALT, NDE was β = 0.077, SE = 0.024, and *p* = 0.002. In contrast, light or moderate drinking did not have a direct effect on mean PD but exhibited an indirect effect through BMI, fasting glucose, and ALT. In Model 1, which includes BMI, NIE was β = 0.009, SE = 0.002, and *p* < 0.001. In Model 2, which includes fasting glucose, NIE was β = 0.003, SE = 0.001, and *p* = 0.015. In Model 3, which includes ALT, NIE was β = 0.007, SE = 0.002, and *p* < 0.001. The contributions of BMI, fasting glucose, and ALT as mediators of the association between light or moderate drinking and mean PD were 25.1%, 8.9%, and 18.9%, respectively. Fasting glucose partially mediated the association between heavy drinking and mean PD (NIE: β = 0.021, SE = 0.009, *p* = 0.023), which corresponded to 32.7% of the proportions mediated.

**Table 3:** Associations among alcohol consumption, mean PD, and three potential mediators in mediation models.

Exposure	Mediator	Outcome	Total effect		NDE (Alcohol→mean PD)		NIE (Alcohol→mediator→mean PD)		
			β (SE)	*p* value	β (SE)	*p* value	β (SE)	*p* value	PM (%)
Model 1									
Alcohol consumption	BMI	Mean PD							
No			Ref		Ref		Ref		
Light/moderate			0.037 (0.016)	0.019	0.028 (0.016)	0.075	0.009 (0.002)	<0.001	25.1
Heavy			0.072 (0.022)	0.001	0.061 (0.024)	0.009	0.011 (0.007)	0.102	N.A.
Model 2									
Alcohol consumption	Fasting glucose^‡^	Mean PD							
No			Ref		Ref		Ref		
Light/moderate			0.031 (0.016)	0.050	0.028 (0.016)	0.073	0.003 (0.001)	0.015	8.9
Heavy			0.065 (0.022)	0.004	0.044 (0.023)	0.060	0.021 (0.009)	0.023	32.7
Model 3									
Alcohol consumption	ALT^‡^	Mean PD							
No			Ref		Ref		Ref		
Light/moderate			0.037 (0.016)	0.022	0.030 (0.016)	0.060	0.007 (0.002)	<0.001	18.9
Heavy			0.070 (0.023)	0.002	0.077 (0.024)	0.002	−0.006 (0.006)	0.340	N.A.

^‡^ Log-transformed values were used.

All models included age, sex, number of present teeth, tooth brushing frequency, regular dental visit, and smoking as covariates.

All models included alcohol consumption as exposure and mean PD as the outcome. BMI, fasting glucose, and ALT were included as mediators in the Model 1, 2, and 3, respectively.

PD, pocket depth; NDE, natural direct effect; NIE, natural indirect effect; PM, proportion mediated; β, β-coefficient; SE, standard error; BMI, body mass index; ALT, alanine aminotransferase; Ref, reference; N.A., not applicable.

### 3.4 Additional analyses

We also conducted mediation analyses using the categorized mediators (Supplementary Table S1). Obesity partially mediated the association between light or moderate drinking and mean PD (NIE: β = 0.004, SE = 0.002, *p* = 0.015), which corresponded to 11.1% of the proportion mediated. Heavy drinking indirectly affected mean PD through elevated glucose (NIE: β = 0.018, SE = 0.008, *p* = 0.012), which corresponded to 29.4% of the proportions mediated. However, abnormal ALT and AST levels did not mediate this association (Supplementary Table S1).

Furthermore, additional mediation models were tested using different definitions of periodontal disease, such as mean CAL and moderate and severe periodontal disease, as outcomes (Supplementary Tables S2–S4). In the results using mean CAL as the outcome, alcohol consumption was not directly associated with mean CAL, but indirectly affected mean CAL through BMI, fasting glucose, and ALT (Supplementary Table S2). Regarding moderate and severe periodontal disease as outcomes, fasting glucose partially mediated the association between heavy drinking and moderate or severe periodontal disease (Supplementary Tables S3 and S4).

We investigated whether fatty liver, another liver function parameter, mediates the association between alcohol consumption and periodontal disease. Fatty liver did not mediate this association (Supplementary Table S5).

## 4 Discussion

Our study revealed that participants with alcohol drinking were more likely to have worse periodontal conditions than non-drinkers. In addition, we found that the association between alcohol consumption and periodontal condition was partially mediated by BMI, fasting glucose, and ALT. Thus, this study extends the findings of previous studies (25, 28–30) concerning alcohol consumption and periodontal disease by demonstrating the mediating effects of multiple factors. These findings suggest that elevated BMI, glucose, and ALT levels have a mediating role in the association between alcohol consumption and periodontal disease.

Our findings regarding the mediating effects of obesity may be explained by some potential mechanisms, as previous studies have confirmed that alcohol consumption contributes to the risk of obesity (31, 32). For example, obesity due to alcohol consumption can be attributed to an increased energy intake from drinking alcohol, which provides 7.1 kcal/g (9). In addition, alcohol can affect some hormones such as leptin and glucagon-like peptide-1, which may contribute to a potential harmful effect on the control of feeding, subsequently leading to a greater intake of food (33). Obesity is a risk factor for periodontal disease (34, 35) due to the secretion of pro-inflammatory cytokines such as TNF-α from adipose tissue, which may induce increased susceptibility to periodontal disease through exacerbated infection with periodontal pathogens and a hyper-inflammatory state (36).

Similarly, there are several potential explanations for our findings regarding elevated glucose as a mediator in the association between light/moderate or heavy drinking and periodontal disease. For example, heavy drinking has been shown to increase the risk of diabetes (37) due to the direct toxic effect of alcohol on pancreatic islet cells, inhibiting insulin secretion and increasing insulin resistance (38). In fact, a previous experimental study found that exposure of pancreatic β-cells to alcohol was associated with reduced insulin secretion (39). This can lead to diabetes, which increases the deposition of advanced glycation end-products in the periodontal tissue and induces the continuous activation of local immune and inflammatory responses, leading to periodontal destruction through increased levels of pro-inflammatory cytokines (40).

Chronic alcohol intake has also long been known to have an effect on liver diseases such as alcoholic hepatitis, fatty liver, and cirrhosis (41). Liver cirrhosis induces immune dysfunction and the excessive activation of pro-inflammatory cytokines, which results in bacterial infection (42). A previous study has shown that a large majority of patients with cirrhosis had periodontal disease, which indicates that cirrhosis may increase the risk of periodontal disease (43). In the present study, continuously elevated ALT levels mediated the association between alcohol consumption and periodontal disease. However, liver abnormalities defined by abnormal levels of ALT did not have an indirect effect on the association between alcohol consumption and periodontal disease. Additionally, the participants in this study did not have severe liver disease, such as liver cirrhosis. These results suggested that elevated ALT owing to alcohol consumption may be associated with periodontal disease, even in the absence of liver abnormalities.

Fatty liver did not mediate the association between alcohol consumption and periodontal disease in this study (Supplementary Table S5), whereas elevated ALT mediated it. Elevated ALT levels are linked to systemic inflammation (44), which may be linked to the worsening of periodontal condition. Fatty liver involves simple steatosis, which is the accumulation of fat in the liver without inflammation, and hepatic steatosis with inflammation as a more advanced stage (45). Participants in our study might not have fatty liver with inflammation, explaining the weak association between fatty liver and periodontal disease in our study.

This study found that heavy drinking had the direct effects on periodontal disease. Previously, heavy drinking has been shown to harmfully affect bone metabolism, leading to alveolar bone absorption and the progression of periodontal disease (7). Additionally, heavy drinking enhances oxidative damage, leukocyte infiltration, and the production of TNF-α in gingival tissues, inducing periodontal deterioration (6). In the present study, the direct effect was found in the PD but not in the CAL (Table 3 and Supplementary Table S1). These results are similar to those reported by Shimazaki et al. (25). This may be because the PD reflects current inflammation in the periodontal tissue, while CAL estimates the lifetime-accumulated periodontal destruction. Additionally, PD tends to be more strongly associated with systemic inflammation than CAL (46), and the direct association between heavy drinking and periodontal disease may be linked to enhanced inflammation.

The association between alcohol consumption and severe periodontal disease tended to be weak, compared to that with the mean PD (Supplementary Table S3). Severe periodontal disease may be linked to other risk factors rather than alcohol consumption. In this study, severe periodontal disease was associated with age and smoking (data not shown). Such risk factors may play a dominant role in severe periodontal disease and may mask the association with alcohol consumption. Given that an association between alcohol consumption and mean PD was observed, alcohol consumption might contribute to an earlier worsening of the periodontal condition.

This study had several limitations. First, the causality between alcohol consumption and periodontal disease could not be determined due to a cross-sectional study. Second, the magnitude of the mediating effects in the association between alcohol consumption and periodontal disease was relatively small. Although this small effect should be interpreted with caution in clinical settings, our findings may provide a valuable input to better understand the mechanisms underlying the association between alcohol consumption and periodontal disease. Third, data on unmeasured potential confounders such as socioeconomic status were not available in this study and may affect our estimates of the association between alcohol consumption and periodontal disease. Third, the periodontal condition was evaluated using partial examination methods described in the NHANES III and did not measure PD on the palate or tongue side, which may have led to an underestimation of periodontal disease. Finally, the study population may not be representative of the general population because this study only included individuals who received workplace health check-ups. Therefore, caution is required when applying these findings to other populations.

In conclusion, this study confirmed that alcohol consumption was positively associated with the periodontal condition among Japanese adults who received workplace health check-ups. This association was partially mediated by several factors such as BMI, fasting glucose, and ALT levels. Future studies should investigate mediators to clarify this association further.

## Data Availability

The datasets presented in this article are not readily available because the data are not publicly available due to ethical restriction. Requests to access the datasets should be directed to Michiko Furuta, mfuruta@dent.kyushu-u.ac.jp.
