# Supplementary material for: Mediating factors associated with alcohol intake and periodontal condition
Source: Front Oral Health. 2025 Apr 24;6:1524772. doi: 10.3389/froh.2025.1524772 (PMC12058805; doi:10.3389/froh.2025.1524772)
Supplement: Supplementary file 1 [file Table1.docx]

Supplementary Material

# Supplementary Tables

| **Supplementary Table 1**. Mediation model including categorized mediators | | | | |  |  |  |  |  |  |
| --- | --- | --- | --- | --- | --- | --- | --- | --- | --- | --- |
| Exposure | Mediator | Outcome | Total effect | | NDE (Alcohol→mean PD) | | NIE (Alcohol→mediator→mean PD) | | | |
|  |  |  | β (SE) | p value | β (SE) | p value | β (SE) | p value | PM (%) |  |
| **Model 1** |  |  |  |  |  |  |  |  |  |  |
| Alcohol consumption | Obesity (BMI≥25.0) | Mean PD |  |  |  |  |  |  |  |  |
| No |  |  | Ref |  | Ref |  | Ref |  |  |  |
| Light/moderate |  |  | 0.033 (0.016) | 0.036 | 0.030 (0.016) | 0.061 | 0.004 (0.002) | 0.015 | 11.1 |  |
| Heavy |  |  | 0.065 (0.022) | 0.003 | 0.059 (0.023) | 0.010 | 0.007 (0.004) | 0.064 | N.A. |  |
| **Model 2** |  |  |  |  |  |  |  |  |  |  |
| Alcohol consumption | Elevated glucose (≥100) | Mean PD |  |  |  |  |  |  |  |  |
| No |  |  | Ref |  | Ref |  | Ref |  |  |  |
| Light/moderate |  |  | 0.030 (0.016) | 0.057 | 0.038 (0.016) | 0.073 | 0.002 (0.001) | 0.015 | N.A. |  |
| Heavy |  |  | 0.063 (0.022) | 0.005 | 0.044 (0.023) | 0.051 | 0.018 (0.008) | 0.012 | 29.4 |  |
| **Model 3** |  |  |  |  |  |  |  |  |  |  |
| Alcohol consumption | Abnormal ALT (males≥30, females≥19) | Mean PD |  |  |  |  |  |  |  |  |
| No |  |  | Ref |  | Ref |  | Ref |  |  |  |
| Light/moderate |  |  | 0.030 (0.016) | 0.060 | 0.030 (0.016) | 0.059 | -0.001 (0.001) | 0.820 | N.A. |  |
| Heavy |  |  | 0.060 (0.022) | 0.007 | 0.061 (0.022) | 0.007 | -0.001 (0.001) | 0.826 | N.A. |  |
| **Model 4** |  |  |  |  |  |  |  |  |  |  |
| Alcohol consumption | Abnormal AST (≥35) | Mean PD |  |  |  |  |  |  |  |  |
| No |  |  | Ref |  | Ref |  | Ref |  |  |  |
| Light/moderate |  |  | 0.030 (0.016) | 0.056 | 0.029 (0.016) | 0.063 | 0.001 (0.001) | 0.165 | N.A. |  |
| Heavy |  |  | 0.061 (0.022) | 0.006 | 0.060 (0.023) | 0.008 | 0.001 (0.002) | 0.698 | N.A. |  |
| All models included age, sex, number of present teeth, tooth brushing frequency, regular dental visit, and smoking as covariates. | | | | | | | | | | |
| All models included alcohol consumption as exposure and mean PD as the outcome. Obesity, elevated glucose, abnormal ALT and AST were included as mediators in the Model 1, 2, 3, and 4respectively. | | | | | | | | | | |
| PD, pocket depth; NDE, natural direct effect; NIE, natural indirect effect; PM, proportion mediated; β, β-coefficient; SE, standard error; BMI, body mass index; ALT, alanine aminotransferase; AST, asparate aminotransferase; Ref, reference; N.A., not applicable. | | | | | | | | | | |

| **Supplementary Table 2.** Mediation model including mean CAL as the outcome | | | | | | |  |  | |  |  |  |
| --- | --- | --- | --- | --- | --- | --- | --- | --- | --- | --- | --- | --- |
| Exposure | Mediator | Outcome | Total effect | | | NDE (Alcohol→mean CAL) | | | NIE (Alcohol→mediator→mean CAL) | | | |
|  |  |  | β (SE) | p value | β (SE) | | p value | β (SE) | | p value | PM (%) |  |
| **Model 1** |  |  |  |  |  | |  |  | |  |  |  |
| Alcohol consumption | BMI | Mean CAL |  |  |  | |  |  | |  |  |  |
| No |  |  | Ref |  | Ref | |  | Ref | |  |  |  |
| Light/moderate |  |  | 0.030 (0.025) | 0.227 | 0.022 (0.025) | | 0.380 | 0.008 (0.003) | | 0.001 | 27.8 |  |
| Heavy |  |  | 0.072 (0.022) | 0.001 | 0.046 (0.035) | | 0.190 | 0.009 (0.010) | | 0.394 | N.A. |  |
| **Model 2** |  |  |  |  |  | |  |  | |  |  |  |
| Alcohol consumption | Fasting glucose^†^ | Mean CAL |  |  |  | |  |  | |  |  |  |
| No |  |  | Ref |  | Ref | |  | Ref | |  |  |  |
| Light/moderate |  |  | 0.026 (0.025) | 0.300 | 0.024 (0.025) | | 0.325 | 0.001 (0.002) | | 0.383 | N.A. |  |
| Heavy |  |  | 0.050 (0.034) | 0.136 | 0.019 (0.034) | | 0.570 | 0.031 (0.012) | | 0.009 | 61.4 |  |
| **Model 3** |  |  |  |  |  | |  |  | |  |  |  |
| Alcohol consumption | ALT^†^ | Mean CAL |  |  |  | |  |  | |  |  |  |
| No |  |  | Ref |  | Ref | |  | Ref | |  |  |  |
| Light/moderate |  |  | 0.031 (0.025) | 0.229 | 0.024 (0.025) | | 0.345 | 0.007 (0.003) | | 0.016 | 22.3 |  |
| Heavy |  |  | 0.055 (0.034) | 0.110 | 0.063 (0.036) | | 0.079 | -0.008 (0.009) | | 0.363 | N.A. |  |
| All models included age, sex, number of present teeth, tooth brushing frequency, regular dental visit, and smoking as covariates. | | | | | | | | | | | | |
| All models included alcohol consumption as exposure and mean CAL as the outcome. BMI, fasting glucose, and ALT were included as mediators in the Model 1, 2, and 3, respectively. | | | | | | | | | | | | |
| ^†^ Log-transformed values were used. | | | | | | | | | | | | |
| CAL, clinical attachment level; NDE, natural direct effect; NIE, natural indirect effect; PM, proportion mediated; β, β-coefficient; SE, standard error; BMI, body mass index; ALT, alanine aminotransferase; Ref, reference; N.A., not applicable. | | | | | | | | | | | | |

| **Supplementary Table 3**. Mediation model including severe periodontal disease as the outcome | | | | | | |  |  |  |
| --- | --- | --- | --- | --- | --- | --- | --- | --- | --- |
| Exposure | Mediator | Outcome | Total effect | | NDE (Alcohol→severe periodontal disease) | | NIE (Alcohol→mediator→severe periodontal disease) | | |
|  |  |  | β (SE) | p value | β (SE) | p value | β (SE) | p value | PM (%) |
| **Model 1** |  |  |  |  |  |  |  |  |  |
| Alcohol consumption | BMI | Severe periodontal disease |  |  |  |  |  |  |  |
| No |  |  | Ref |  | Ref |  | Ref |  |  |
| Light/moderate |  |  | 0.004 (0.006) | 0.487 | 0.003 (0.006) | 0.620 | 0.001 (0.001) | 0.054 | N.A. |
| Heavy |  |  | 0.020 (0.009) | 0.026 | 0.016 (0.009) | 0.078 | 0.004 (0.003) | 0.176 | N.A. |
| **Model 2** |  |  |  |  |  |  |  |  |  |
| Alcohol consumption | Fasting glucose^†^ | Severe periodontal disease |  |  |  |  |  |  |  |
| No |  |  | Ref |  | Ref |  | Ref |  |  |
| Light/moderate |  |  | 0.004 (0.006) | 0.535 | 0.004 (0.006) | 0.577 | 0.001 (0.001) | 0.334 | N.A. |
| Heavy |  |  | 0.019 (0.006) | 0.535 | 0.014 (0.009) | 0.128 | 0.005 (0.002) | 0.035 | 26.7 |
| **Model 3** |  |  |  |  |  |  |  |  |  |
| Alcohol consumption | ALT^†^ | Severe periodontal disease |  |  |  |  |  |  |  |
| No |  |  | Ref |  | Ref |  | Ref |  |  |
| Light/moderate |  |  | 0.004 (0.006) | 0.588 | 0.003 (0.006) | 0.642 | 0.001 (0.001) | 0.497 | N.A. |
| Heavy |  |  | 0.019 (0.009) | 0.041 | 0.003 (0.006) | 0.030 | -0.002 (0.003) | 0.419 | N.A. |
| All models included age, sex, number of present teeth, tooth brushing frequency, regular dental visit, and smoking as covariates. | | | | | | | | | |
| All models included alcohol consumption as exposure and severe periodontal disease as the outcome. BMI, fasting glucose, and ALT were included as mediators in the Model 1, 2, and 3, respectively. | | | | | | | | | |
| ^†^ Log-transformed values were used. | | | | | | | | | |
| NDE, natural direct effect; NIE, natural indirect effect; PM, proportion mediated; β, β-coefficient; SE, standard error; BMI, body mass index; ALT, alanine aminotransferase; Ref, reference; N.A., not applicable. | | | | | | | | | |

| **Supplementary Table 4**. Mediation model including moderate periodontal disease as the outcome | | | | | | |  |  |  |
| --- | --- | --- | --- | --- | --- | --- | --- | --- | --- |
| Exposure | Mediator | Outcome | Total effect | | NDE (Alcohol→moderate periodontal disease) | | NIE (Alcohol→mediator→moderate periodontal disease) | | |
|  |  |  | β (SE) | p value | β (SE) | p value | β (SE) | p value | PM (%) |
| **Model 1** |  |  |  |  |  |  |  |  |  |
| Alcohol consumption | BMI | Moderate periodontal disease |  |  |  |  |  |  |  |
| No |  |  | Ref |  | Ref |  | Ref |  |  |
| Light/moderate |  |  | 0.018 (0.016) | 0.248 | 0.015 (0.016) | 0.348 | 0.003 (0.002) | 0.023 | 18.8 |
| Heavy |  |  | 0.033 (0.021) | 0.116 | 0.032 (0.022) | 0.146 | 0.001 (0.006) | 0.865 | N.A. |
| **Model 2** |  |  |  |  |  |  |  |  |  |
| Alcohol consumption | Fasting glucose^†^ | Moderate periodontal disease |  |  |  |  |  |  |  |
| No |  |  | Ref |  | Ref |  | Ref |  |  |
| Light/moderate |  |  | 0.017 (0.016) | 0.291 | 0.016 (0.016) | 0.310 | 0.001 (0.001) | 0.518 | N.A. |
| Heavy |  |  | 0.031 (0.021) | 0.139 | 0.019 (0.021) | 0.383 | 0.012 (0.006) | 0.045 | 39.5 |
| **Model 3** |  |  |  |  |  |  |  |  |  |
| Alcohol consumption | ALT^†^ | Moderate periodontal disease |  |  |  |  |  |  |  |
| No |  |  | Ref |  | Ref |  | Ref |  |  |
| Light/moderate |  |  | 0.019 (0.016) | 0.222 | 0.017 (0.016) | 0.292 | 0.003 (0.002) | 0.119 | N.A. |
| Heavy |  |  | 0.034 (0.021) | 0.101 | 0.040 (0.022) | 0.066 | -0.005 (0.006) | 0.330 | N.A. |
| All models included age, sex, number of present teeth, tooth brushing frequency, regular dental visit, and smoking as covariates. | | | | | | | | | |
| All models included alcohol consumption as exposure and moderate periodontal disease as the outcome. BMI, fasting glucose, and ALT were included as mediators in the Model 1, 2, and 3, respectively. | | | | | | | | | |
| ^†^ Log-transformed values were used. | | | | | | | | | |
| NDE, natural direct effect; NIE, natural indirect effect; PM, proportion mediated; β, β-coefficient; SE, standard error; BMI, body mass index; ALT, alanine aminotransferase; Ref, reference; N.A., not applicable. | | | | | | | | | |

| **Supplementary Table 5**. Mediation model including fatty liver as a mediator and different definitions of periodontal condition as the outcome | | | | | | | | | |
| --- | --- | --- | --- | --- | --- | --- | --- | --- | --- |
| Exposure | Mediator | Outcome | Total effect | | NDE (Alcohol→periodontal condition) | | NIE (Alcohol→fatty liver→periodontal condition) | | |
|  |  |  | β (SE) | p value | β (SE) | p value | β (SE) | p value | PM (%) |
| **Model 1** |  |  |  |  |  |  |  |  |  |
| Alcohol consumption | Fatty liver | Mean PD |  |  |  |  |  |  |  |
| No |  |  | Ref |  | Ref |  | Ref |  |  |
| Light/moderate |  |  | 0.031 (0.016) | 0.052 | 0.030 (0.016) | 0.064 | 0.002 (0.001) | 0.066 | N.A. |
| Heavy |  |  | 0.062 (0.022) | 0.005 | 0.061 (0.023) | 0.007 | 0.002 (0.002) | 0.444 | N.A. |
| **Model 2** |  |  |  |  |  |  |  |  |  |
| Alcohol consumption | Fatty liver | Mean CAL |  |  |  |  |  |  |  |
| No |  |  | Ref |  | Ref |  | Ref |  |  |
| Light/moderate |  |  | 0.025 (0.025) | 0.317 | 0.024 (0.025) | 0.338 | 0.001 (0.001) | 0.303 | N.A. |
| Heavy |  |  | 0.046 (0.034) | 0.168 | 0.043 (0.034) | 0.200 | 0.003 (0.003) | 0.276 | N.A. |
| **Model 3** |  |  |  |  |  |  |  |  |  |
| Alcohol consumption | Fatty liver | Severe periodontal disease |  |  |  |  |  |  |  |
| No |  |  | Ref |  | Ref |  | Ref |  |  |
| Light/moderate |  |  | 0.002 (0.007) | 0.760 | 0.002 (0.007) | 0.750 | -0.001 (0.001) | 0.773 | N.A. |
| Heavy |  |  | 0.017 (0.009) | 0.067 | 0.016 (0.009) | 0.089 | 0.017 (0.009) | 0.067 | N.A. |
| All models included age, sex, number of present teeth, tooth brushing frequency, regular dental visit, and smoking as covariates. | | | | | | | | | |
| All models included alcohol consumption as exposure and fatty liver as a mediator. Mean PD, CAL, and severe periodontal disease were included as outcomes in the Model 1, 2, and 3, respectively. | | | | | | | | | |
| PD, pocket depth; CAL, clinical attachment level; NDE, natural direct effect; NIE, natural indirect effect; PM, proportion mediated; β, β-coefficient; SE, standard error; BMI, body mass index; ALT, alanine aminotransferase; Ref, reference; N.A., not applicable. | | | | | | | | | |

# Supplementary Figures


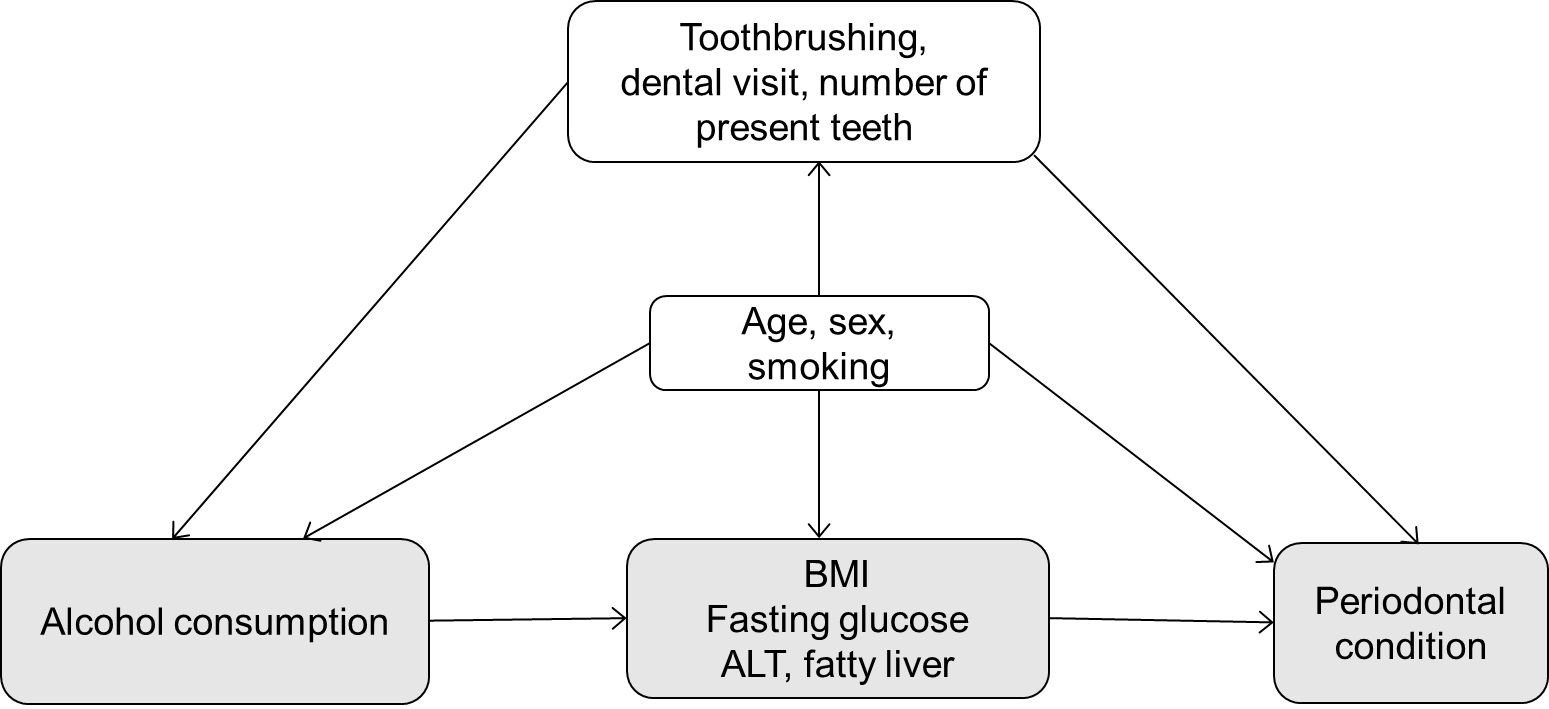


**Supplementary Figure 1.** Directed acyclic graph (DAG) representing presumed the association among alcohol consumption and periodontal condition. Nodes represent variables and arrows represent causal associations. Gray-colored nodes represent the causal effect of interest, which is periodontal condition as outcome, alcohol consumption as exposure, and BMI, fasting glucose, ALT, and fatty liver as mediator.
